# Point-Contact Spectroscopy in Bulk Samples of Electron-Doped Cuprate Superconductors

**DOI:** 10.3390/ma16247644

**Published:** 2023-12-14

**Authors:** Angela Nigro, Anita Guarino, Antonio Leo, Gaia Grimaldi, Francesco Avitabile, Paola Romano

**Affiliations:** 1 Physics Department “E. R. Caianiello”, University of Salerno, 84084 Fisciano (Salerno), Italy; anigro@unisa.it; 2 CNR-SPIN, c/o University of Salerno, 84084 Fisciano (Salerno), Italy; anita.guarino@spin.cnr.it (A.G.); antonio.leo@spin.cnr.it (A.L.); gaia.grimaldi@spin.cnr.it (G.G.); francesco.avitabile@spin.cnr.it (F.A.); 3 Department of Sciences and Technologies, Sannio University, 82100 Benevento, Italy

**Keywords:** electron-doped cuprates, point-contact spectroscopy, tunneling

## Abstract

Point-contact spectroscopy was performed on bulk samples of electron-doped high temperature superconductor Nd_2−x_Ce_x_CuO_4−δ_. The samples were characterized using X-ray diffraction and scanning electron microscopy equipped with a wavelength-dispersive spectrometer and an electron backscatter diffraction detector. Samples with Ce content x = 0.15 showed the absence of spurious phases and randomly oriented grains, most of which had dimensions of approximately 220 µm^2^. The low-bias spectra in the tunneling regime, i.e., high-transparency interface, exhibited a gap feature at about ±5 meV and no zero-bias conductance, despite the random oriented grains investigated within our bulk samples, consistent with most of the literature data on oriented samples. High-bias conductance was also measured in order to obtain information on the properties of the barrier. A V-shape was observed in some cases, instead of the parabolic behavior expected for tunnel junctions.

## 1. Introduction

It is well known that point-contact spectroscopy (PCS) can give insight into the size and nature of a superconducting energy gap. This technique has been widely used on conventional as well as new generation superconductors [1,2,3,4,5,6]. By changing the pressure between a tip made of normal metal (N) and a superconducting sample (S), it is possible to obtain different types of contacts, i.e., different electrical transport regimes that depend on the nature of the interface. Both quasiparticle NIS junctions (here, I stands for insulating barrier) and NS contacts obtained through PCS can be used to study the energy gap in superconductors [7]. By changing the tip position on the surface of a polycrystalline sample, with random oriented crystallites, different tunneling directions with respect to the crystallographic orientation of the crystalline grain can be investigated, thus providing information on the anisotropy of the superconducting order parameter and the possible presence of nodes such as in d-wave symmetry of the superconducting order parameter.

Among high-temperature superconductors, electron-doped copper oxides, with the chemical formula RE_2−x_Ce_x_CuO_4_ (RE = Pr, Nd, Sm, La), have also been investigated using PCS. As in the case of hole-doped high-T_c_ cuprates, the phase diagram of electron-doped cuprates shows a well-known ‘dome’ shape with the highest T_c_ at a doping level of about 0.15, but with a superconducting window much narrower and an antiferromagnetic phase that persists at higher doping levels [8,9,10,11]. Several techniques have been used to investigate the electronic properties of electron-doped superconductors, including angle-resolved photoemission spectroscopy (ARPES), tunneling spectroscopy, magnetic penetration depth measurements, and transport measurements. ARPES measurements were used to reveal the presence of multiple bands in the electronic structure [12,13,14,15,16,17,18,19,20,21,22]. In particular, the overdoped region is characterized by a large hole-like Fermi surface that undergoes a Fermi surface reconstruction at intermediate doping levels with the presence of electron and hole pockets. In contrast, only electron pockets are present in the underdoped region.

Superconducting and normal state properties of electron-doped cuprates have been extensively investigated using resistivity, magnetotransport, and Hall effect measurements. In particular, the sign change of the Hall coefficient measured in cerium over-doped samples has been described as evidence of the presence of two types of charge carriers, electrons and holes, as also supported by ARPES experiments, and the appearance of superconductivity has been related to the presence of holes [23,24,25,26,27,28,29,30].

As revealed by tunneling experiments and magnetic penetration depth measurements, the symmetry of the superconducting order parameter in electron-doped cuprates has been more controversial, unlike hole-doped cuprates, for which the pairing symmetry is predominantly d-wave. Several experimental data on n-type samples are consistent with an order parameter having d-wave symmetry [13,14,19,20,22,31,32]. However, many tunneling experiments showed no evidence of a zero-bias conductance peak (ZBCP), expected for a d-wave superconducting system, suggesting a pairing symmetry of the s-wave type. Furthermore, PCS experiments on Pr_2−x_Ce_x_CuO_4_ at different Ce doping levels have shown evidence of ZBCP in underdoped samples, suggesting a change in symmetry from d- to s-wave with increasing doping [28,32,33,34,35]. The absence of ZBCP has also been attributed to the coexistence of antiferromagnetic and superconducting orders [36]. Magnetic penetration depth measurements on Pr_2−x_Ce_x_CuO_4_ and La_2−x_Ce_x_CuO_4_ (RE = Pr, La) films at different Ce content also suggested a d-wave to anisotropic s-wave transition across optimal doping [37,38]. A two-band model with d-wave pairing symmetry and different doping-dependent amplitudes has been successfully used to describe the magnetic penetration depth measurement and Raman experiments on electron-doped cuprate superconductors [26,27]. Unlike hole-doped cuprates, there is no clear consensus on the characteristic of the gap function in the electron-doped cuprates family. Indeed, special gap functions and/or two-band models have been invoked to explain experimental data.

Among cuprates, electron-doped oxides do not show a strong pseudogap at the antinodal region. This constitutes an advantage in the investigation of the superconducting gap, which is relatively small. In the past, various studies have shown that the superconducting pairing strength is close to a weak coupling regime, at least in the optimally doped and overdoped region [31,39]. The study of the superconducting density of states with the energy gap is performed through low-bias conductance measurements. On the other hand, the normal state can be also investigated by means of high-bias conductance measurements. In the past, linear background conductance has been observed in many HTS compounds and related to the normal density of states of cuprate superconductors. This behavior has been ascribed to the interlayer coupling mechanism between CuO_2_ planes [40].

As highlighted in the aforementioned studies, electron-doped compounds show some similarities but also several differences in their properties compared to hole-doped compounds. In recent years, these disparities have made electron-doped cuprates particularly intriguing materials since they may provide some additional insight into the microscopic mechanism of superconductivity in cuprates [41,42,43,44,45]. Indeed, despite the intense work carried out on hole-doped cuprates, this mechanism is still an open question.

In this work, we report PCS measurements on optimally doped Nd_2−x_Ce_x_CuO_4−δ_ bulk samples in order to investigate the possibly angular dependence of the gap function. The point contact technique combined with samples in polycrystalline form, characterized by randomly oriented single-crystal grains, provides the possibility of exploring the behavior of the tunneling conductance along different directions with respect to the crystallographic orientation of grains, allowing for investigation of the possible anisotropy of the superconducting order parameter. Most of the experimental tunneling data on electron-doped compounds reported in the literature was carried out on oriented samples, films, or single crystals, which only allowed for some specific tunneling directions to be investigated. The differential conductance of tunnel junctions with different contact resistances was acquired. Differential conductance at low bias showed the presence of superconducting gap structures at ±5 meV, consistent with values already reported in the literature. The absence of the peak at zero-bias expected for an order parameter with nodes, as observed in the hole-doped compounds, is also discussed. The temperature dependence of the conductance showed well-defined gap structures up to 15 K; above this temperature, the conductance flattened and the gap closed, as is expected for a superconducting gap. This paper is organized as follows: Section 2 describes the experimental details of fabrication and characterization of our bulk samples of Nd_2−x_Ce_x_CuO_4−δ_ compound. In Section 3, the Blonder, Thinkam, and Klapwijk (BTK) theory of point-contact spectroscopy is briefly summarized. Section 4 reports our results on point contact measurements and the related discussion and conclusions.

## 2. Sample Characterization

In Nd_2−x_Ce_x_CuO_4−δ_ samples, the superconductivity occurs in a narrow range of Ce doping, 0.10–0.24, with the highest T_c_ = 24 K at a doping level of about 0.15.

Unlike hole-doped HTS, for electron-doped compounds, not only is doping required, but a proper oxygen-reducing process is essential to observe superconductivity [8,9,46]. The role of this reduction process is a long-standing issue on electron-doped high-temperature superconductors. Indeed, the as-grown samples do not show superconductivity, and it is necessary to perform thermal treatment in a low-oxygen environment and at high temperatures to drive the samples into a superconducting state. This reduction process affects the amount of oxygen atoms and their distribution in the crystalline structure and significantly impacts the transport and magnetic properties of the samples, transforming them from antiferromagnetic semiconductors to superconducting metals [47,48,49,50,51,52,53].

The electron-doped compounds crystallize in a T’ crystal structure with one square planar copper–oxygen CuO_2_ layer in between charge reservoir layers, characterized by the absence of oxygen ions at apical sites (above and below CuO_2_ layers).

Recently, experimental and theoretical findings on as-grown oxygen-deficient Nd_2−x_Ce_x_CuO_4−δ_ films suggested a structural reorganization involving oxygens ions at apical positions that remodulates the electronic band structure. No apical oxygens, or too many of them, stabilize strong antiferromagnetic correlations, whereas an intermediate number of apical oxygens suppresses antiferromagnetic correlations and allows for the availability of holes at the Fermi level. This is consistent with the presence of both holes and electrons in the superconducting samples, as revealed by many experiments [54,55,56,57,58].

Bulk samples of Nd_1.85_Ce_0.15_CuO_4_ (NCCO) were synthesized using the standard solid-state reaction technique. To remove carbon dioxide and moisture, precalcination was performed on Nd_2_O_3_ and CeO_2_ for about 10 h at 950 °C. Afterwards, a stoichiometric amount of 4 N purity Nd_2_O_3_, CeO_2_, and CuO were weighed and grinded in an agate mortar using acetone as a binding agent. After calcinations in air at 1020 °C for 24 h, the samples were reground, pressed into pellets, and sintered in air at 1150 °C for 24 h. Finally, a reduction process at 900 °C for 30 h in argon atmosphere was carried out to observe the superconducting transition [59].

**Figure 1:**
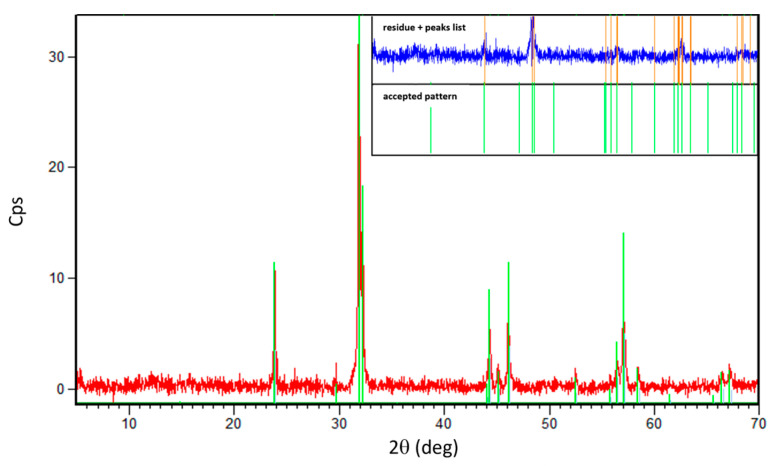
Diffractogram of our NCCO sample after the reduction process (red line). The inset shows the diffraction identifications panel usingX’Pert Highscore software: the accepted pattern (green lines) corresponds to the chart ICDD 01-080-1645 containing the crystallographic parameters of a bulk sample of Nd_1.85_Ce_0.15_CuO_4_. The upper panel of the inset shows the peaks list (orange lines) selected from the raw data and the residue pattern (blue line) obtained from the difference between orange and green patterns.

X-ray diffraction measurements confirmed the crystalline quality and the phase purity of the final sample. Figure 1 reports an example of diffractogram of the powder obtained from our NCCO after all the thermal treatments: by using X’Pert HighScore software version 4.1, we found a perfect match of our diffractogram with the reflections of the tetragonal NCCO phase with space group I4/mmm (ICDD 01-080-1645) and cerium content of 0.15.

**Figure 2:**
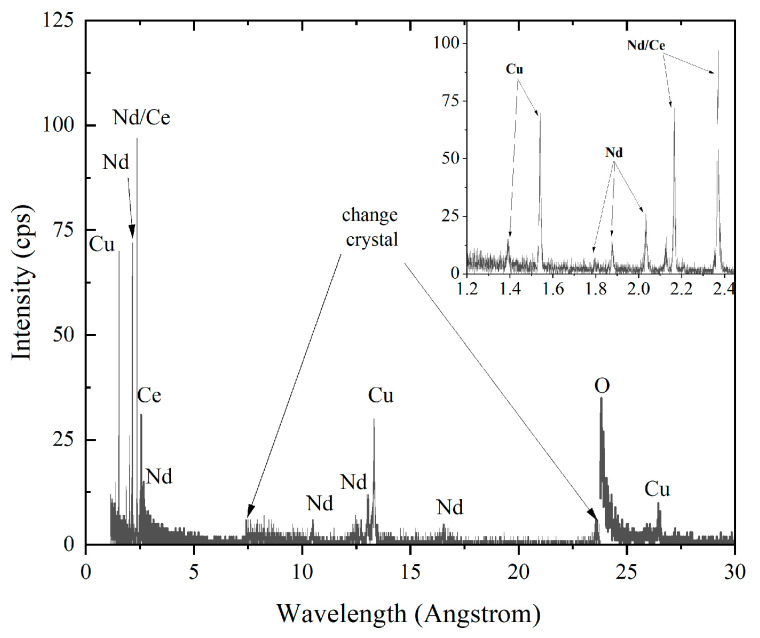
WDS trace scan elements in the wavelength range 1.14–30 Å for an NCCO sample. In the inset, a zoom in the range from 1.2 to 2.4 Å is shown. All the peaks are first- or higher-order Bragg reflections of X-rays characteristic of Nd, Ce, Cu, and O.

The chemical composition of the samples was studied using a scanning electron microscope (SEM, Oxford LEO-Evo50) equipped with a wavelength-dispersive spectrometer (WDS, Oxford INCA Wave detector, INCA version 18d)) and the measurements confirmed a cerium content of 0.15. The possible presence of some spurious elements was checked using WDS scans on the whole surface at a range of wavelengths going from 1.14 to 175 Å. The results obtained for one of the samples are shown in Figure 2 for a reduced wavelength range, from 1.14 to 30 Å. In this range, all the peaks can be attributed to X-ray reflections from Nd, Ce, Cu, and O, which are elements of the compound. The scan performed on all the samples in the whole range, i.e., up to 175 Å, revealed no trace of spurious elements within the experimental error.

**Figure 3:**
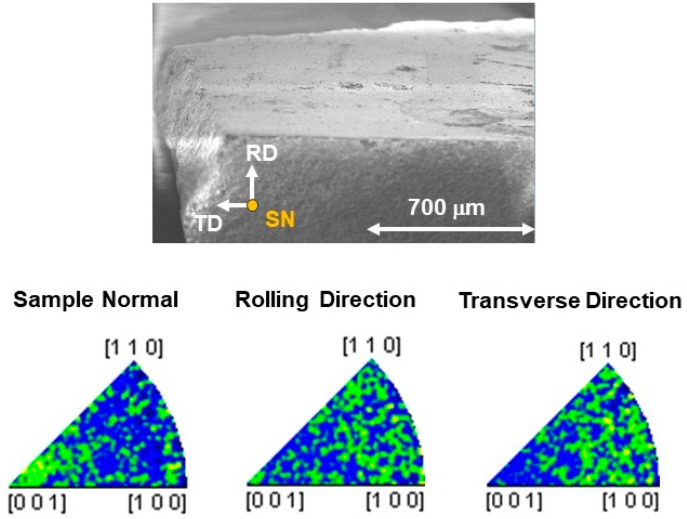
SEM image of a NCCO sample is shown. SN, RD, and TD represent the sample direction reference with respect to which the orientation of the crystal cells is reported. The inverse pole figures plotted with crystal directions as the axes of the figure, for each sample direction, denote the polycrystalline nature of the sample. Yellow areas correspond to the highest intensities, blue areas to the lowest ones.

A grain analysis was performed on these pure and stoichiometric samples by using electron backscattered diffraction (EBSD) measurements, carried out with a SEM LEO-EVO 50 equipped with an INCA Crystal 300 EBSD system. The electron beam scanned a flat selected area of the sample, properly prepared using the metallographic technique (diamond pastes down to 1 μm), and the diffracted X-rays were collected in a pattern on a phosphor screen showing the so-called Kikuchi bands. The sample was mounted on a special stub, tilted 70° with respect to the horizontal plane, where three directions were defined as a reference for the orientation of the sample: the sample normal (SN), perpendicular to the sample surface, and the rolling direction (RD) and transverse direction (TD) in the sample plane, perpendicular to each other and with respect to the SN. Using its database, the software identified what phase the pattern corresponded to and the crystalline cell orientation for each pixel of the SEM image. The different orientations were highlighted according to a color-coded stereographic triangle related to the phase added in the database. In Figure 3, a SEM image of a metallographically prepared (diamond paste down to 1 µm) polycrystalline sample in the EBSD configuration is shown together with the orientation references. The inverse pole figures reported below for each reference denote the polycrystalline nature of the sample: in fact, the random distribution of the intensities in the three pole figures indicates that no preference orientations were detected.

**Figure 4:**
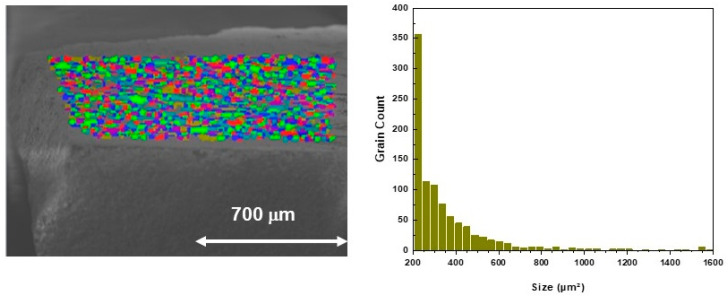
On the left, SEM image of the sample with its superimposed orientation map. Each grain has a different color, grains with the same color have the same orientation. On the right, a grain size plot from which it follows that the majority of grains of this sample has dimensions of about 220 μm^2^.

**Figure 5:**
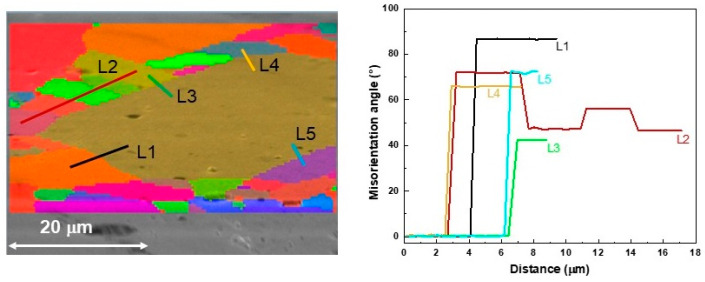
On the left, grain map calculated with a misorientation angle threshold of 5° and superimposed on an SEM image at high magnification. Each grain is assigned a discrete color to differentiate it from the neighboring grains. On the right, changes in misorientation angles for the different profile scans indicated on the grain map.

Figure 4 reports the SEM image of the same sample with its superimposed orientation map: several grains were detected with different orientations, i.e., different colors on the map, according to the color-code key of the NCCO phase. The histogram on the right plots the number of grains detected in the orientation map as a function of their dimensions; it resulted that the majority of grains had size of about 220 μm^2^. In order to evaluate the misorientation among the several grains, a grain map was analyzed. Figure 5 shows such a map of the sample around one of the larger grains. In this map, each grain is assigned a discrete color to differentiate it from the neighboring grains. The right panel of Figure 5 represents the changes in misorientation angles for the different profile scans highlighted on the map. The sharp profiles suggest that even grains with a larger misorientation result strongly interconnected without exhibiting spurious phases at the interfaces, as also confirmed using compositional SEM analysis (not reported here).

**Figure 6:**
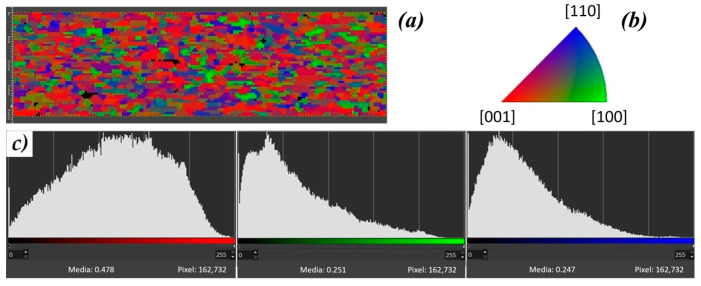
(**a**) Orientation map along the normal direction of a sample where each color corresponds to a specific orientation of the crystalline cell for that grain taking into account the reference color key reported in panel (**b**) for the NCCO tetragonal crystalline structure. (**c**) Distributions of the pixels with a certain color intensity selecting different channels. The horizontal axis in the histograms is the light level of color: 0 means zero light and 255 means maximum light.

From the EBSD analysis, it is clear that the sample is polycrystalline, and it is not possible to identify a preferential orientation of the grains (see Figure 3). Looking at the orientation map along the normal direction reported in Figure 6a, each color in the map corresponds to a specific orientation of the crystalline cell ([001], [100], or [110]) for that grain taking into account the reference color key (Figure 6b). Even if there is no preferred orientation, a graphical analysis of the orientation map image along the normal direction could provide some information on the number of pixels with a certain color. By selecting the red, green, or blue channel in the graphic software, it is possible to represent, on a histogram, the color intensity related to the particular channel with a media value, as shown in Figure 6c. On average, the red histogram shifted towards higher mean intensity, which can be interpreted as a greater number of grains having a significant component in the [001] direction, compared to the other orientations.

## 3. Theory of NS Contacts

When a point contact is realized between a superconductor (S) and a normal metal (N), a small contact area forms, giving rise to an NS junction. Andreev’s reflections (ARs) may occur when an electron in N incident at the interface has an energy level lower than the S energy gap, forming a Cooper pair that can enter in S, leaving a hole in N. The AR is then a retroreflection of a hole of opposite spin and velocity but momentum equal to the incident electron. From an experimental point of view, the formation of Andreev bound states are seen in the differential conductance spectra as a peak at zero bias. In fact, a net charge transfer of 2*e* occurs from N to S at the high-transparency (i.e., transmission coefficient) limit; the conductance of the junction, when all electrons incident at the NS interface with energy lower than the superconducting gap are subject to AR, is twice the conductance of the normal state [60,61]. The transparency of the interface can, however, be changed by changing the distance and/or pressure tip/sample in this way, giving rise to different electrical transport regimes. It is so possible to make a combination of point contact and barrier-type junctions by using PCS, realizing different kinds of contacts. In particular, when the transparency is low enough, a tunnel junction is obtained, which is usually indicated as an NIS junction where I stands for the insulating barrier.

The tunnel current in junctions made using two different materials can be written as [7]:

$$I(V)=G_{NN}\int_{-\infty }^{\infty }\rho_{1}(E)\rho_{2}(E+eV)\left[f(E)-f(E+eV)\right]dE$$

Here, *ρ*_1_(*E*) and *ρ*_2_(*E*) are the quasiparticle DOS in the two electrodes, *f*(*E*) is the Fermi–Dirac distribution function that describes thermal smearing, and *G_NN_* = *R_NN_* is the junction conductance when both electrodes are in the normal state.

For a normal metal–insulator–superconductor (NIS) junction where electrode 1 is a normal metal, *ρ*_1_(*E*) = 1, and electrode 2 is a superconductor with a DOS *ρ*(*E*), in the limit *T* = 0 *K*, the tunneling conductance d*I*/d*V* becomes:

$${\left.\frac{dI}{dV}\right|}_{SN}\equiv G_{SN}=G_{NN}\int_{-\infty }^{\infty }\rho (E)\left[\frac{-\partial }{\partial E}f(E+eV)\right]dE=G_{NN}\rho (E)={\left.\frac{dI}{dV}\right|}_{NN}\rho (E)$$

Here, we assumed *E* = *eV*. The bracketed function is sharply peaked with half-width 3.5 *k_B_T*, so in the limit of *T* = 0 *K*

$$\rho =\frac{G_{SN}}{G_{NN}}$$

The superconducting DOS at *T* = 0 *K* can thus be obtained from the ratio of the tunneling conductance when one of the electrodes is in the superconducting state to the tunneling conductance when both electrodes are in the normal state.

The theory of superconductivity by Bardeen, Cooper, and Schrieffer (BCS) [62] was used to predict a characteristic DOS structure:

$$\begin{array}{ll} \rho (E)=\frac{\left|E\right|}{{\left(E^{2}-\Delta^{2}\right)}^{1/2}}\;\;\; & \left|E\right|\geq \Delta \\ \rho (E)=0\;\;\; & \left|E\right|<\Delta \end{array}$$

where Δ is the energy gap. An exact determination of *ρ(E)* requires a measurement of normal state (background) conductance *G_NN_*. While *G_SN_* is readily measured at *T* << *T_C_*, the measurement of *G_NN_* can be difficult. It is possible to introduce a magnetic field to quench the superconductivity, or to raise the temperature above *T_c_*. However, a background shape can be drawn from high-bias conductance and used to normalize the measured overall conductance.

Both quasiparticle NIS junctions and NS contacts obtained through PCS can be used to study the energy gap in superconductors [7]. For the general case of a junction between N and S, with or without an insulating barrier, the differential conductance can be expressed by means of the Blonder, Thinkam, and Klapwijk (BTK) [63] theory:

$$G_{NS}=\frac{4}{4+Z^{2}}\int_{-\infty }^{+\infty }\left[1+A(E)-B(E)\right]\cdot \frac{\partial f(E+eV)}{\partial (eV)}dE$$

The *Z* parameter modulates the interface between N and S. The case *Z* = 0 corresponds to an NS junction, with a completely transparent barrier, in which the dominant mechanism responsible for the transport current is the Andreev process. In this case, Andreev reflections (ARs) can constitute the predominant process at the interface and the shape of the conductance is different, showing the so-called zero-bias anomaly (ZBA), hallmark of an AR.

On the other hand, *Z* > 1 represents a junction with a low transparent barrier, corresponding to a dominant tunneling current flowing through the junction—in other words, an NIS junction. *A(E)* represents the Andreev reflection probability and *B(E)* represents the normal reflection probability.

**Figure 7:**
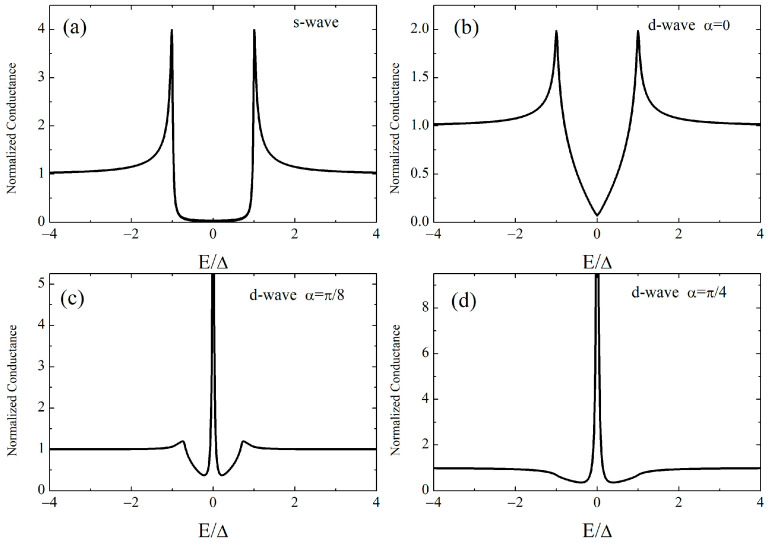
Normalized conductance at T = 0 K and with Z = 5 in the case of isotropic s-wave symmetry (**a**) and d-wave symmetry with (**b**) α=0, (**c**) α=π/8,  and (**d**) α=π/4.

The BTK theory can be extended to the case of unconventional superconductors with different symmetries of the order parameter (OP). In this case, the normalized conductance at *T* = 0 *K* is written as:

$$G\left(E\right)=\frac{\int_{-\frac{\pi }{2}}^{+\frac{\pi }{2}}\sigma \left(E,\varphi \right)\cos\varphi d\varphi }{\int_{-\frac{\pi }{2}}^{+\frac{\pi }{2}}\sigma_{N}\left(\varphi \right)\cos\varphi d\varphi }$$

$$\sigma \left(E,\varphi \right)=\sigma_{N}\frac{1+\sigma_{N}{\left|\gamma_{+}\left(E\right)\right|}^{2}+\left(\sigma_{N}-1\right){\left|\gamma_{+}\left(E\right)\gamma_{-}\left(E\right)\right|}^{2}}{{\left|1+\left(\sigma_{N}-1\right)\gamma_{+}\left(E\right)\gamma_{-}\left(E\right)\right|}^{2}}$$

$$\sigma_{N}\left(\varphi \right)={\left(1+\frac{Z^{2}}{\cos^{2}\varphi }\right)}^{-1};\gamma_{\pm }\left(E\right)=\frac{1}{{∆}_{\pm }}\left(E-\sqrt{E^{2}-{{∆}_{\pm }}^{2}}\right)$$

where φ is the incident angle of the quasiparticle and *E* = *eV*, with *V* being the bias voltage. For an isotropic s-wave order parameter ${\;∆}_{+}={∆}_{-}=∆$, while in the case of d-wave symmetry ${∆}_{\pm }=∆\cdot \cos\left(2\alpha \mp 2\varphi \right)$, where α is the angle between the crystallographic a-axis and the normal to the tunneling interface. Figure 7 shows the normalized conductance at *T* = 0 *K* and with *Z* = 5 (tunneling regime) in the case of isotropic s-wave symmetry, panel (a), and for d-wave symmetry with α=0, α=π/8 , and α=π/4, panel (b), (c), and (d), respectively. The ZBA could also be originated from a d-wave order parameter, as shown in Figure 7c,d.

In order to account for the often-observed asymmetric background conductance, a generalization of the BTK theory was proposed, including three regimes: direct tunneling between N and S, tunneling through the localized states into S, resonance between direct tunneling and tunneling via localized states.

A two-channel tunneling model including these three regimes was proposed by Fogelstrom et al. to generalize the BTK theory of point contact NS conductance. In this model, tunneling can occur directly from N to S, or via localized states giving rise to a Fano resonance [64] that generates asymmetry in the high-bias conductance.

## 4. Experimental Results and Discussion

### 4.1. Low-Bias Conductance

PCS consists of making contact by pressing a metal tip onto a superconducting sample to form a small contact area, i.e., an NS interface (or junction). By changing the distance and/or pressure between tip and sample, different electrical transport regimes can be realized that depend on the transparency of the interface. In particular, a quasiparticle tunnel is obtained from low junction transparency, while the point contact corresponds to the case in which the barrier is low (high interface transparency). In real experiments, an intermediate regime can also be observed depending on the intensity of interface transparency. In this work, PCS was performed by pushing a Au tip on the top of NCCO bulk samples in a liquid helium bath. The samples were electrically characterized by using a four-probe configuration, with a current supply source meter (Keithley mod. 2400, Cleveland, OH, USA) and a nanovoltmeter (Keithley mod. 2182A, Cleveland, OH, USA). The current–voltage characteristics were recorded and differential conductances numerically calculated. Contacts with different resistances were measured by changing the pressure and distance between tip and sample, as well as by changing the tip position on the sample.

**Figure 8:**
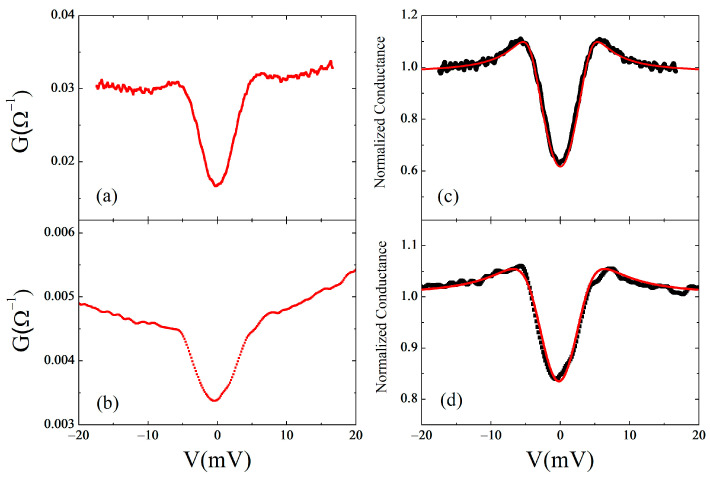
Differential conductance for junctions with resistances (**a**) 45 Ω and (**b**) 150 Ω; normalized conductance for the junctions of panel (**a**,**b**) compared with the BTK model for a d-wave order parameter—solid red line. (**c**) α = 0, Δ = 4.5 meV, Z = 2.2, and Γ = 1.8; (**d**) α = 0, Δ = 4.5 meV, Z = 1.5, and Γ = 3.2.

The low-bias differential conductances at 4.2 K are shown in Figure 8a,b for two contacts with a resistance of about 45 Ω and 150 Ω, respectively. The spectra show a feature at about ±5 meV, and this was routinely observed for contacts with resistances above 45 Ω. The conductance decreases around zero bias, showing the behavior expected for tunnel junctions. This is an indication that the contact formed between the tip (N) and the sample (S) is in the tunneling regime, i.e., the transparency of the interface NS is low, and a natural tunnel barrier is present. The results are consistent with the literature data, reported on bulk, single crystals, and films [9,65,66].

We estimated the junction size using the Sharvin formula $R_{N}=4\rho l/\left(3\pi d^{2}\right)$, in which the normal resistance of the junction is related to the contact dimension *d* and to the product ρl, with *ρ* being the resistivity and l the mean free path of the carriers of the superconducting material. Since a very high anisotropy is found in NCCO single crystals with an out-of-plane resistivity *ρ_c_* five orders of magnitude larger than the in-plane resistivity *ρ_ab_*, we assume *ρ* = *ρ_ab_* and for the 2D system $\rho l=hc_{o}/e^{2}k_{F}$, where $c_{o}=0.6\;nm$ is the distance between two adjacent CuO_2_ layers in NCCO unit cell and $k_{F}\approx 3\;{nm}^{-1}$ is the Fermi wave vector for the NCCO material. [9,67,68,69,70]. Based on the Sharvin formula, we found the junction size to be *d* = 7 nm and *d* = 4 nm, respectively. This confirms that the point contact is in the ballistic regime [71], in which the size of the junction is smaller than the mean free path in the superconductor (*d* << ℓ).

**Figure 9:**
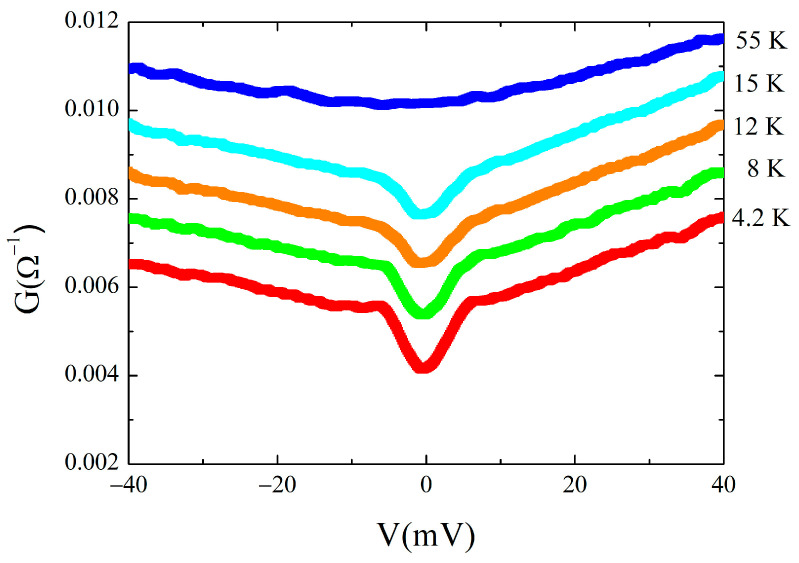
Conductance temperature dependence for the 150 Ω junction.

The shape and the temperature dependence of the ±5 meV feature allowed us to identify it with the superconducting gap. For one of the measured contacts, in fact, the temperature dependence of the conductance is shown in Figure 9. The gap is well visible up to 15 K, and above this temperature, the conductance flattens and the gap closes as expected considering its superconducting nature.

A slightly increasing background conductance was observed for biases higher than the gap voltage, which is analyzed in the following section.

In a polycrystalline sample, consisting of randomly oriented grains, by changing the position of the tip on the sample surface, it is possible to investigate the tunneling conductance along different tunneling directions with respect to the crystallographic orientation of the crystalline grains. However, in a polycrystalline sample, it is not possible to associate each measured conductance spectrum to a particular tunneling direction. Nevertheless, in the case of an anisotropic order parameter, possibly with nodes, different types of conductance spectra are expected on different grains within the polycrystalline sample. No zero-bias conductance was observed in our tunneling measurements and tunneling spectra of the type shown in Figure 8 were always measured. This is consistent with most tunneling results on oriented samples, which show no differences in conductance spectra along [001], [100], and [110].

Figure 8c,d shows a comparison between the experimental normalized conductance curves and the BTK model for a d-wave order parameter, solid red lines, for the junctions of Figure 8a,b. We used α = 0, Δ = 4.5 meV, and Z = 2.2 and Z = 1.5 for the junctions of Figure 8a,b respectively.

The broadening parameter Γ related to a reduction in quasiparticle lifetime introduced by Dynes et al. was also introduced in the conductance model by substituting E with E + iΓ. Generally, Γ can be used to take into account the possible presence of inelastic scattering processes due to the poor interface quality, energy gap distribution, and, in our case, to account for the small thermal smearing due to the low but finite temperature [72,73]. In Figure 8a, we used Γ = 1.8, which for the ratio Γ/Δ provided a value of 0.4. Commonly, Γ/Δ < 0.5 is found for the best experimental conductance curves. In Figure 8b, we used Γ = 3.2, which gives Γ/Δ ≈ 0.7, consistent with the high resistance of the junction [74].

The results could be indicative of a dominant current along the antinodal direction of the sample in case of order parameters with nodes or of a weak angle dependence of the gap function (constant for s-wave symmetry). The absence in the conductance of a zero-bias peak as well as no dependence on the tunneling direction in most tunneling experiments was attributed to a nonmonotonic d-wave order parameter. Also, the peculiar structure of the Fermi surface close to the optimal Ce doping value with two pockets, one around (π,0) and another around (π/2,π/2), suggested an s-wave component within a two-band model scenario. Furthermore, the dirty limit character of electron-doped materials, with a mean free path lower than the BCS coherence, could account for tunneling data features [14,15,28,75,76].

It was proposed that in electron-doped cuprates, antiferromagnetic (AF) and superconducting (SC) orders may coexist. The BTK formula was generalized to include an AF coupling [36,77,78]. When the AF order Φ = 0, a zero-energy state is responsible for the zero-bias conductance peak (ZBCP) widely observed in hole-doped d-wave cuprate superconductors. When Φ ≠ 0, the zero-energy state disappears, and the energy of the existing state is always finite. It has been argued that there is no ZBCP when AF and SC orders coexist, and for this reason, it often does not show up in electron-doped cuprates.

### 4.2. High-Bias Conductance

**Figure 10:**
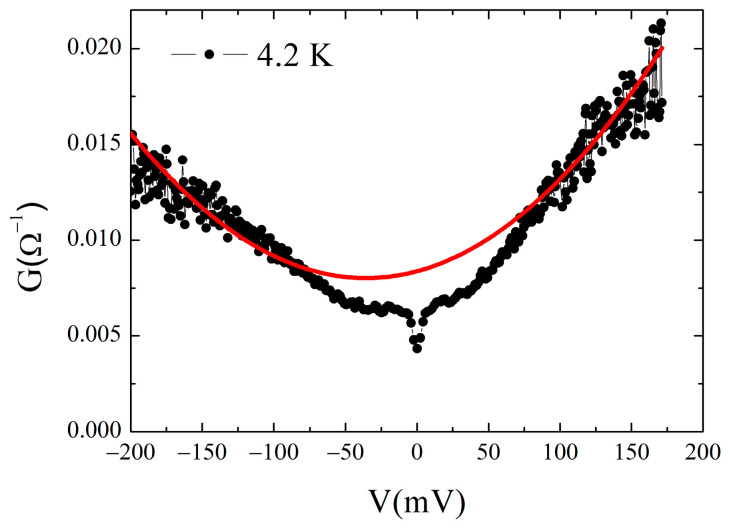
High-bias conductance: parabolic background for the 85 Ω junction. Red line is the fit to the parabolic conductance expected by the Simmons theory.

We also performed measurements for voltages much higher compared to the peak position. In some cases, a parabolic background is well visible, as shown in Figure 10 at 4.2 K.

In a tunnel junction, the high bias behavior is usually related to the barrier properties. When no additional current channels other than pure tunneling are contributing, the Simmons theory provides a parabolic conductance at voltages well above the energy gap value [79]:

$$G(V)=G_{0}(1+bV+3\gamma V^{2})$$

with $G_{0}=\left(3.16\;\cdot 10^{10}\;\cdot \;{\bar{\phi }\;}^{1/2}/\;∆s\right)\;exp(-1.025\;\cdot \;∆s\;\cdot \;{\bar{\phi }\;}^{1/2})$ and $\gamma =0.0115\;\cdot \;{∆s}^{2}\;/\bar{\;\phi }$, where $\bar{\phi }$ is the average height, in eV, and ∆s the thickness, in Å, of the barrier. The linear term bV accounts for the barrier asymmetry [80]. From the fitting parameters of the high-bias parabola, the average height and the thickness of the barrier can be calculated. For the conductance curve shown in Figure 10, at voltages $\left|V\right|>100\;mV$, the resulting barrier’s parameters are $\bar{\phi }\;\sim 7\;eV$ and ∆s ~ 10 Å. This behavior has also been observed in PCS performed on c-axis-oriented NCCO thin films [81].

**Figure 11:**
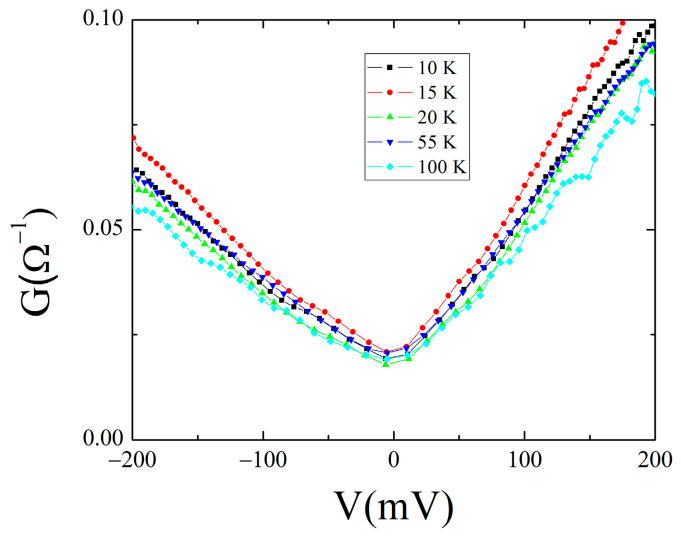
High-bias conductance: linear background for the 7.5 Ω junction.

In some cases, a definite V-shape is observed, as shown in Figure 11.

A linear differential conductance has been often observed for HTCS-based junctions with a larger slope for tunneling along the c-axis [82]. A similar behavior has been reported regarding directional in-plane tunneling PCS performed on single crystals of optimally doped Nd_1.85_Ce_0.15_CuO_4-y_, by driving a Au tip toward the crystal along the [100] and [110] direction [15,28].

Considering the polycrystalline nature of our sample and the anisotropic properties of the NCCO compound, the different slopes of the background conductance could be related to different tunneling directions with respect to the orientation of the crystalline grains that may contribute to the overall shape of the conductance.

The linear background can be caused by an intrinsic mechanism like density of states effects [40] as well as from inelastic tunneling from a broad distribution of scattering states as a result of nonstoichiometric oxygen in natural barrier of HTCS-based junctions [83].

On the other hand, in addition to direct tunneling, even localized states can contribute to the conductance, for instance, coming from impurity and/or imperfections. In this case, a Fano resonance may provide an asymmetry in the conductance, like that observed in our high-bias data [64].

In conclusion, PCS was performed on bulk samples of electron-doped high-temperature superconductor NCCO. The samples were characterized using a SEM equipped with a WDS for compositional measurements and with an EBSD detector for the analysis of grain orientation distribution. The X-ray diffraction technique was also used for structural characterization.

Considering the polycrystalline nature of our samples, different types of conductance spectra are expected on different grains within the sample in the case of an anisotropic order parameter. Junctions in the tunneling regime were obtained with superconducting gap structures at ±5 meV. The conductance curves were compared with the BTK model for a d-wave order parameter and the only differences observed in low-bias conductance curves were related to the value of the Γ/Δ ratio. Furthermore, no zero-bias conductance peak was observed. The results could be indicative of a dominant current along the antinodal direction of the sample in case of order parameter with nodes or of a weak angle dependence of the gap function. The high-bias conductances were also measured and different background conductance was observed, probably related to different tunneling directions with respect to the orientation of the crystalline grains. In particular, a linear background was often observed, indicative of intrinsic or extrinsic mechanisms.

## Data Availability

The data presented in this study are available on reasonable request from the corresponding author.
